# Implementation Fidelity in Early Intervention for Eating Disorders—A Multisite Pilot Study

**DOI:** 10.3390/bs15111521

**Published:** 2025-11-08

**Authors:** Lucy Hyam, Lucy Gallagher, Zoe Tsivos, Sarah Macnab, Ben Kirton, Emily Palmer, Eóin Killackey, Karina L. Allen, Ulrike Schmidt

**Affiliations:** 1 Centre for Research in Eating and Weight Disorders, Department of Psychological Medicine, Institute of Psychiatry, Psychology and Neuroscience, King’s College London, London SE5 8AB, UK; 2 The Willows Centre for Health, Greater Manchester Mental Health NHS Foundation Trust, Lords Avenue, Salford M5 5JR, UK; 3 Wiltshire Community Eating Disorders Service, Oxford Health NHS Foundation Trust, Wiltshire SN8 3HL, UK; 4 Oxfordshire Adult Community Eating Disorders Service, Oxford Health NHS Foundation Trust, Oxford OX3 7JX, UK; 5 Sussex Eating Disorder Service, Sussex Partnership NHS Foundation Trust, Worthing BN2 3EW, UK; 6 Orygen, 35 Poplar Rd., Parkville, VIC 3052, Australia; 7 Centre for Youth Mental Health, The University of Melbourne, 35 Poplar Rd., Parkville, VIC 3052, Australia; 8 Centre for Population Health, Helse-Bergen HF, Haukeland Universitetssjukehus, Postboks 1400, 5021 Bergen, Norway; 9 Eating Disorders Outpatients Service, Maudsley Hospital, South London and Maudsley NHS Foundation Trust, Denmark Hill, London SE5 8AZ, UK

**Keywords:** eating disorders, early intervention, fidelity, service development, implementation

## Abstract

First Episode Rapid Early Intervention for Eating Disorders (FREED) is an early intervention service model and care pathway for young people aged 16–25 with a recent-onset eating disorder. This multi-site study aimed to evaluate the feasibility of a newly developed implementation fidelity tool and observe patterns of fidelity to FREED. Six eating disorder services across England took part in an assessment. Data were collected from 242 patient referrals between January and June 2025 and via semi-structured interviews with FREED staff. Total fidelity scores were calculated alongside two component scores (rapid access to the service and early intervention care package), and inter-rater agreement was assessed. The tool was feasible to use across multiple services, time efficient, aligned with existing workflows, and demonstrated high inter-rater agreement. The average fidelity score across services was 72% (‘medium fidelity’). The average component scores were 57% for rapid access targets (‘not satisfactory’) and 77% for early intervention care package (‘medium fidelity’). Rapid access target scores were highly variable across services (20–87%), whereas care package scores were more consistent (72–82%). Additional sustained resource augmentation is needed to improve model adherence and facilitate consistent access to high-quality early intervention for eating disorders, including support to meet rapid access targets.

## 1. Introduction

First Episode Rapid Early Intervention for Eating Disorders (FREED) is the leading early intervention model for eating disorders (EDs). It is a care pathway and service model for young people (aged 16–25) with any ED, in the early stages of illness (duration of untreated ED [DUED] ≤ 3 years). FREED aims to offer rapid access to evidence-based treatment that is tailored to the unique needs of this population. For example, the FREED care package includes a focus on malleability and reversibility of brain and bodily changes early on, providing a hopeful and empowering outlook for the young person, as well as a rationale for early nutritional change. It also includes greater involvement of family or close others than conventional ED treatment for adults, flexible and needs-based support for transitions (e.g., to university or between child/adolescent and adult services), and guidance around social media use. The full FREED model is described in detail by Fukutomi et al. (2020). Since completing national rollout in 2023, FREED has been implemented in 54 NHS Trusts across England and is being adapted internationally, including in Canada (FREEDCan), Australia (EmergED), and the Netherlands (Vibes).

FREED’s implementation has been guided by key implementation science frameworks, including RE-AIM (reach; effectiveness/efficacy; adoption; implementation; maintenance) for planning and initial scale-up (Allen et al., 2020). However, there has been limited systematic assessment of implementation fidelity, i.e., how faithfully the model is implemented in routine practice (Carroll et al., 2007; Mowbray, 2003). Measurements of fidelity typically assess both the organisation of the service and quality of programme delivery. Repeated fidelity assessments are particularly valuable for monitoring adherence to evidence-based models (Williams et al., 2021), promoting parity of access to high-quality care across different services. Fidelity measurement can also explain why interventions may not replicate their original outcomes when scaled or applied to ‘real world’ routine clinical settings (Breitenstein et al., 2010). Furthermore, fidelity measurement can support quality improvement and identify areas where support or training is needed, particularly when findings are shared with clinicians, supervisors, and their teams (Mueser et al., 2019).

Service and treatment fidelity are essential for maintaining the integrity and effectiveness of implementation (Cromarty, 2024). In EDs, existing studies have primarily focussed on treatment-level fidelity, i.e., whether treatment models are delivered as intended (Couturier et al., 2021; Waller & Turner, 2016). This research is limited in number and adherence measures are deemed resource-intensive, costly, and infrequently used (Peyser et al., 2021). Research on service model fidelity (i.e., ensuring the service is managed and delivered as intended) is also limited, though some studies have examined programme-level fidelity, for example, in child and adolescent mental health services (Sippy et al., 2025).

To support fidelity during FREED’s national scaling, implementation support was available including local support from the Health Innovation Network (Health Innovation Network, 2024) and monthly implementation supervision with the FREED national team—clinicians and researchers based at South London and Maudsley NHS Foundation Trust (SLaM) and King’s College London (KCL). During this period, a single evaluation of implementation fidelity measured adherence to access time targets and care package components across four services over 12 months (Richards et al., 2021). Although adherence to access targets improved following FREED implementation, it was recognised that ongoing measurement of fidelity to the FREED service model and care package was necessary to avoid drift. Potential differences in patterns of fidelity across services may lead to variability in outcomes and inconsistent service delivery across regions. Moreover, sustained increases in service demand following the COVID-19 pandemic may affect adherence to FREED access times (Gallagher et al., 2025).

To address this need, we recently developed the first fidelity tool for early intervention EDs, designed for FREED or similar models (Hyam et al., 2025). The tool was based on similar fidelity measurement in early intervention psychosis services (Addington, 2021; Addington et al., 2020; Williams et al., 2021). Its development was supported by an iterative consultation process within the FREED national team. The core aims, functions, principles, and components of FREED and early intervention for EDs were reviewed and transformed into 35 testable items across two key components.

The first component assesses rapid access to the service via wait time targets for assessment (within 2 weeks of referral) and treatment (starting within 4 weeks of referral). The second component investigates adherence to the FREED care package and other important early intervention items (e.g., family involvement, adaptations to treatment, data collection of core early intervention items such as DUED). Both treatment and service model fidelity are important to FREED implementation, however, the tool has a specific focus on service model fidelity.

We piloted the tool in a London-based FREED service with patient referral data spanning July 2024–June 2025 and with data from interviews with members of this FREED team (Hyam et al., 2025). The tool showed initial feasibility as it was largely aligned with existing infrastructure for collecting data and was time efficient. Overall fidelity scores were in the lower range at both timepoints (63% at time 1 and 60% at time 2). Regarding component-specific scores, rapid access targets demonstrated lowest fidelity, with scores of ‘not satisfactory’ at both time points (30% at time 1 and 20% at time 2). For adherence to the care package, scores were in the medium fidelity range (76% and 75% at time 1 and 2, respectively). These results demonstrated the utility of fidelity assessment in identifying implementation gaps and areas for improvement. For example, this assessment demonstrated that investment is urgently required to speed up access to evidence-based treatment, as has been echoed in the wider ED literature (Viljoen et al., 2024).

The lower to moderate fidelity scores from the pilot assessment provide a clear rationale for continued development and use of the FREED fidelity tool to monitor service delivery across England. In early intervention psychosis services, longitudinal fidelity assessment, coupled with ongoing consultation with services, resulted in the achievement of ‘superior’ fidelity in all groups of services by the third visit/assessment (Williams et al., 2021). To assess feasibility across diverse settings and guide refinement, the FREED tool now requires testing in a broader sample beyond one pilot site. This will help identify wider, and ultimately national, patterns of fidelity for service planning and improvement. The current study extends our initial single-site pilot assessment and evaluates (1) the tool’s feasibility across multiple FREED services and (2) patterns of fidelity across services. As this study is exploratory, no specific hypotheses were formulated.

## 2. Materials and Methods

### 2.1. Design and Sample

Building on prior work detailing the conceptualisation, development, and longitudinal single-site pilot assessment of our early intervention EDs fidelity tool, the present study reports a multisite evaluation of this tool. This incorporates a cross-sectional analysis of routinely collected clinical data alongside interview data from each service.

Six FREED services across England were recruited for this study. One service (SLaM) participated in the initial feasibility testing. Their data for the same time period are re-included in this assessment to allow for broader comparison. The remaining five services were selected using a mix of self-selection and convenience sampling. Services were invited based on data availability for the study period and to try and capture diverse areas across England. To maintain confidentiality, the additional FREED services are deidentified and denoted as Site A–E.

### 2.2. Fidelity Tool

**Figure 1:**
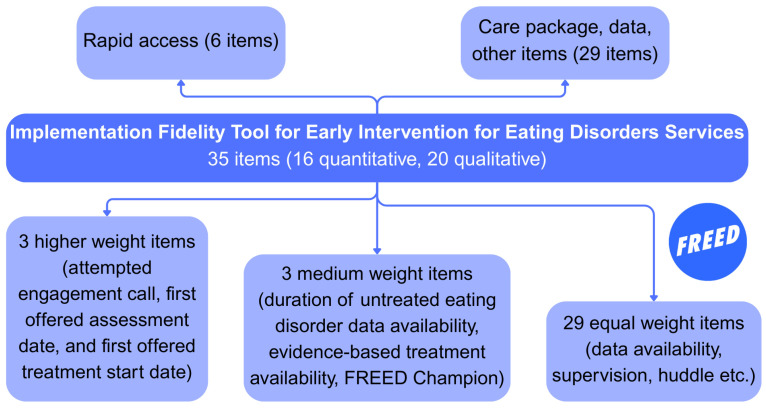
Overview of the FREED Fidelity Tool.

The fidelity tool was developed following guidance from toolkits and from examples of fidelity measurement in the early intervention psychosis literature (Addington et al., 2020; Bond et al., 2000; Williams et al., 2021). The tool consists of 35 items assessing adherence to the FREED service model and care package (Figure 1). These 35 items are divisible into two broad components. The first component captures rapid access to the service via six items assessing time from referral to an initial engagement call, assessment, and treatment. The second component contains 29 items evaluating use of the FREED care package such as whether the FREED adaptations to assessment and treatment are used, availability of early intervention data (e.g., DUED on referral), and how family involvement is encouraged in a FREED patient’s journey. This component also captures other service features or components that make for high-quality early intervention (e.g., self-referral, use of and skill in motivational interviewing). A full breakdown of item content, scoring criteria, and weighting allocations is provided in Supplementary Materials S1. Supplementary Materials S2 contains further explanation on the FREED model and each item included in the assessment.

Of the 35 items, 16 are quantitatively assessed using routinely collected FREED data (see Richards et al. (2023) for further detail), and 19 are qualitatively assessed via a semi-structured interview with key staff at each service (interview prompts are available in Supplementary Materials S3). Each item is scored on a 5-point scale ranging from 1 (not satisfactory) to 5 (maximum fidelity). Binary items are scored as 1 (absent) or 5 (present). For example, a score of 5 is given if all intended ages are seen within the FREED pathway (16–25 years old, or 18–25 if adult only service); a score of 1 is given if there is an age restriction to referrals.

Items are weighted to reflect their perceived importance in the FREED model, with rationale provided in Hyam et al. (2025). Weights total 100% with a higher weight (6.82%) first assigned to three items concerning rapid access times. A moderate weight (4.55%) is then assigned to three items assessing DUED data collection, availability of evidence-based treatment for all patients, and the appointment of a suitable FREED Champion (a clinician typically at UK NHS Agenda for Change band 7, such as a psychologist, nurse, or therapist with a significant level of expertise and leadership experience). All remaining items are assigned an equal, lower weight (2.27%). Scores for each item are multiplied by their respective weight and then summed to provide a total fidelity score. Component scores are calculated by dividing item scores by the maximum possible weighted score for that component. Table 1 details the total possible weighted scores and their corresponding fidelity labels.

### 2.3. Data Collection

For the fidelity assessments, quantitative items were scored with data from 242 FREED patient referrals across the six services spanning a six-month period (1 January–30 June 2025). Qualitative items were scored through online, semi-structured interviews conducted with members of the FREED team at each participating service. This was usually the FREED Champion. For one service, the interview was conducted with another member of the FREED mini-team. Interviews were carried out by two researchers, both of whom had experience in FREED implementation and data evaluation. Participants were informed that the fidelity tool was ‘a work in progress’ and that the study aimed to improve the tool rather than evaluate their or their service’s performance. They were encouraged to provide honest responses. Each participating service was provided with a feedback report detailing their score, areas of strength, and areas for improvement. An example feedback report is available from the authors. Services were invited to provide feedback and discuss any queries with the interviewing researchers.

### 2.4. Ethical Approval

When joining the FREED network, all services sign an Operational Agreement for data sharing. Patient data are deidentified before being shared with the FREED national team for processing. Only pooled data are shared for the purpose of the ongoing evaluation of the effectiveness of FREED. Patients are informed about data sharing and given the opportunity to opt out (see Richards et al. (2023) for further detail). As the fidelity assessment aligns with how data are usually shared and processed, no further ethical approval was required. Service participation in this study was optional.

### 2.5. Analysis

Quantitative data from FREED data trackers were analysed by the authors using RStudio version 4.3.1 (R Core Team, 2024). Item scores were weighted and exported to Microsoft Excel. Interview data were scored, weighted, and added to the spreadsheets separately. Weighted scores were summed to generate overall fidelity scores, as well as summary scores for the two components per service and across all services.

Inter-rater agreement was assessed for each item using linear and quadratic weighted Cohen’s kappa coefficients (Li et al., 2023). To evaluate overall agreement for total fidelity scores, we calculated the intraclass correlation coefficient (ICC3k for 2 fixed raters). Reliability statistics were computed in RStudio using the irr and psych packages (R Core Team, 2024).

## 3. Results

### 3.1. Feasibility

Six FREED services across England participated in this study, contributing a total of 242 referrals between January and June 2025. Detailed demographics of patient referrals are not available due to data sharing agreements requiring data sets to be deidentified and protected characteristics excluded. The average age of the sample was 19.75 years (*SD* = 2.49, *n* = 240/242). Diagnoses included 71 individuals with anorexia nervosa, 42 with other specified feeding or eating disorders (12 with atypical anorexia nervosa, 1 with PD, and the remainder without a specific OSFED sub-type specified), 18 with bulimia nervosa, 7 with binge eating disorder, and 3 with avoidant/restrictive food intake disorder (*n* = 141/242^1^). Of five services that were invited, four participated and one did not respond. One additional service volunteered to participate and SLaM were re-included. These six services represent five adult community ED services and one all-age ED service in the South East, South West, and North West regions of England and in London, spanning both rural and urban catchment areas. FREED is currently primarily embedded within adult and all-age services; thus the sample reflects the context in which the model is most commonly delivered. Therefore, the tool was feasible for use across a mix of service structures, and geographic and demographic contexts. Further, the data required to assess almost half of the items are data that are already routinely collected by FREED services and processed by the FREED national team. Interviews for qualitative items lasted between 60 and 90 min. The tool seemed largely acceptable to the FREED staff involved in assessments. One of the FREED Champions involved in an assessment said:

“I really like the fidelity tool and how it has been developed and piloted. Having received our report, I can see how it is going to influence improvement in our FREED service, even within resource limitations, by helping us know where to focus that resource and increase motivation as well.”

### 3.2. Fidelity Scores

**Figure 2:**
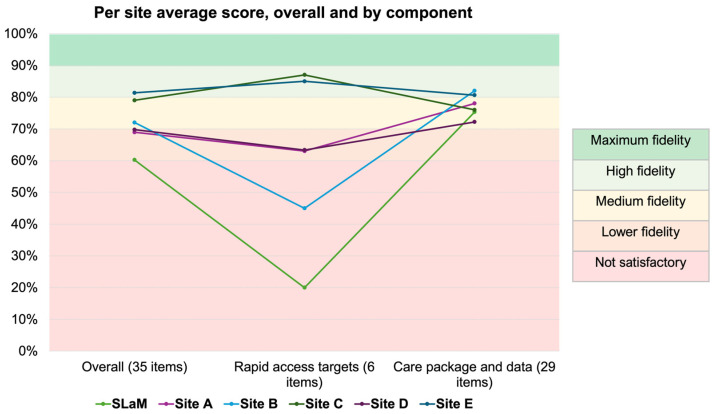
Average Score per Service Across Overall and Component Measures.

The distribution of referrals and fidelity assessment results, overall and by service, are presented in Table 2 and Figure 2.

In this first multisite fidelity assessment across 6 FREED services, the average overall fidelity score was 72%, corresponding to ‘medium fidelity’ (Table 1). For the separate components, a score of 57% was calculated for rapid access targets (component one), classified as ‘not satisfactory’, and a score of 77% was calculated for the care package component (component two), which falls within the ‘medium fidelity’ range. Overall fidelity ranged from 60% (lower fidelity) to 81% (high fidelity) across the services. Scores for rapid access targets were particularly variable, ranging from 20% (not satisfactory) to 87% (high fidelity). In contrast, care package and data scores were more consistent, ranging from 72% (medium fidelity) to 82% (high fidelity).

### 3.3. IRR

Weighted kappa values indicated ‘almost perfect’ agreement between raters, with scores for each service ranging from 0.93 to 0.99 using a quadratic weighting scheme and from 0.86 to 0.98 using a linear weighted scheme (Landis & Koch, 1977). Intraclass correlation coefficients using the ICC(3,k) model demonstrated ‘excellent’ reliability, with values ranging from 0.97 to 1 (Koo & Li, 2016). Reliability scores for all service are reported in Appendix A.

## 4. Discussion

This study aimed to explore the feasibility and utility of the FREED fidelity tool, as well as patterns of FREED model adherence, across six FREED services in England. Findings showed that average fidelity to the FREED model across all participating services was ‘medium’ (72%). However, scores varied considerably, particularly in adherence to the rapid access targets (from 20% to 87%). Care package adherence was more consistent, ranging between ‘medium’ (72%) and ‘high’ fidelity (82%), suggesting these aspects of the model may be more readily integrated into routine practice.

Inter-rater reliability statistics demonstrated consistent scoring between raters, suggesting that the tool can be reliably used by different raters with appropriate training. The tool was also easily used across a range of service contexts (a mix of all-age and adult-only ED services). Together, these findings suggest the tool is feasible and useful, providing a rationale for scaling its use to support the ongoing evaluation of early intervention fidelity.

### 4.1. Fidelity Scores—The Importance of Context

We found major variation in adherence to rapid access targets across the 6 FREED services. This is concerning, as longer waiting times are associated with worsening symptoms and consequent poorer treatment outcomes (Allen et al., 2023). While two services demonstrated high fidelity to FREED’s rapid access targets, two services had ‘lower’ and another two had ‘not satisfactory’ scores for this component. This variation must be understood in the context of increased demand on FREED services during and following the COVID-19 pandemic (Gallagher et al., 2025; Hyam et al., 2023). This demand has not been matched with increased resources. On the contrary, many services had to make so-called ‘efficiency savings’, or experienced recruitment freezes which reduced clinician time (e.g., resulting in a loss of 5 full-time psychological therapists in the SLaM service over the last 2.5 years). This leaves services with a dilemma: whether to prioritise resources to meet FREED access targets or to spread them more broadly to reduce overall service-wide waiting times. Ultimately, our findings add to calls for substantial investment in adult ED services to reduce waiting times, not just in FREED, but for all patients (Viljoen et al., 2024). To increase service capacity, there is also scope for further innovation in how ED treatment is delivered (task-sharing, use of focussed and programme-led interventions (Allen et al., 2025; Wade, 2025) but these innovations cannot fully offset inadequate service resourcing.

In this context, we should also consider the impact of low fidelity scores and ‘not satisfactory’ labels on staff morale. Fidelity assessment must strike a balance between striving for improvement and being supportive, rather than punitive, and recognise the structural limitations staff may face (Aarons et al., 2009). The score labels were modelled on the bandings given by Addington et al. (2020) and Williams et al. (2021) for first episode/early psychosis services and were already adjusted to be more lenient, due to the aforementioned context of resource limitations i.e., a score of <60% equalling the lowest fidelity label compared to <65% in Williams et al. (2021) and <70% in Addington et al. (2020). Nonetheless, further refinement may be needed to reflect the challenges ED services face. A growth-oriented framework, framing scores as stages of development (e.g., ‘developing fidelity’) may shift the focus from criticism to potential.

Facilitating high fidelity requires continued funding (Csillag et al., 2018). Fidelity measurement in the early psychosis youth system in Australia was accompanied by targeted funding for areas with lower fidelity scores, which helped the tool become more readily accepted by services. Moreover, the reinstatement of government funding for the programme and greater programme stability enabled services to improve their adherence to the model (EY et al., 2020). Thus, when fidelity measurement is paired with supportive mechanisms to remediate poor performance, there may be less risk to staff morale and greater engagement with the fidelity measurement process.

### 4.2. Other Patterns of Fidelity

FREED was designed to minimise gatekeeping of ED treatment by removing weight-based or diagnostic severity thresholds to access treatment (Brown et al., 2018). This fidelity assessment returned scores between lower and medium fidelity for accessibility of FREED to all diagnoses and ED presentations, raising concerns about whether this goal has been achieved. This was primarily driven by an inability for services to accept avoidant/restrictive food intake disorder (ARFID) diagnoses on the FREED pathway, due to service commissioning restrictions, despite FREED being intended for all ED diagnoses. Early intervention is critical for improving outcomes in ARFID and NHS policy mandates access to treatment within 4 weeks for children and young people below age 18 (Archibald & Bryant-Waugh, 2023). However, our assessment demonstrates the gap in accessing early intervention treatment for emerging adults with ARFID presenting to all-age and adult services.

The item assessing availability of DUED data also scored low across services. DUED is a potentially modifiable factor influencing outcomes in EDs and thus a core metric for early intervention research (Austin et al., 2021; Schmidt et al., 2016). However, DUED data can be difficult to obtain since patients often struggle to pinpoint the onset of specific symptoms. Further, calculating it can be time-consuming for clinicians. These barriers limit our ability to evaluate the impact of DUED on treatment outcomes, or the value of this criterion for FREED eligibility. Addressing this gap in data collection is therefore crucial for clinical practice and research advancement. The Eating Disorders Clinical Research Network (EDCRN) is a new initiative which will standardise data collection (including DUED for FREED patients) across participating ED services in the United Kingdom (Jewell et al., 2025). Whilst this standardisation will help integrate routine measurement of DUED into daily clinical practice, it may not fully resolve problems with missingness.

Community awareness and outreach activities, together with promoting diversity and inclusion of under-served groups, are two further areas for improvement. This was also highlighted in our first single-service fidelity assessment. Help-seeking is low in ED populations, particularly for under-served groups such as people from minoritised communities, those of lower SES, males, and those in larger bodies (Wilkins et al., 2025). FREED services are therefore encouraged to proactively engage with their local community to increase the visibility and reach of early intervention and reduce stigma—for example, by raising awareness among general practitioners (GPs), who are often the first point of contact but receive limited ED training (Ayton & Ibrahim, 2018; Lebow et al., 2021). Additional outreach could include partnerships with universities and third-sector organisations. In parallel, ED services must adapt treatment for under-served individuals, such as young people affected by poverty and food insecurity (Bryson et al., 2025). FREED Champions need supervision, support and protected time to fulfil the demands of their role and ultimately to improve equitable, accessible, timely care for all.

### 4.3. Future Work

The FREED fidelity tool is likely to require ongoing iterative refinement. For example, further consultation and formal feedback should be sought with staff and service users. The fidelity tool was developed by the FREED national team who are well-versed with the model and its implementation. This team includes senior academics and clinicians, researchers, and former FREED Champions. Some have lived ED experience. However, open questions include whether all the items in the assessment are necessary or if any aspects of early intervention are missing, if the weighting distribution of items is suitable, and, what an optimal assessment period for routine fidelity measurement is (currently, a six-month period has been used). As discussed above, we also need to consider whether the labels associated with fidelity scores are appropriate for ED services. Answering these questions and optimising the tool requires input from a wider range of FREED clinicians and those with lived ED experience.

One aim when developing the fidelity measurement was to ensure practicality within the time and resource constraints of NHS ED services. As such, a key strength is that the assessment requires online meetings only and data that are routinely collected and shared by FREED staff. However, the evolving landscape of data-sharing presents challenges for future fidelity assessment. The end of national FREED funding means researchers will no longer have access to routine service-level FREED data. The gold standard for fidelity research is in-person, on-site assessment (Becker et al., 2015; Bond & Drake, 2020), which could allow raters to observe team processes and physical aspects of the environment such as accessibility and inclusivity. However, this approach is highly resource intensive. An alternative and potentially more efficient route is using self-report methods, similar to those used in early intervention psychosis services; however, self-rated fidelity scores tend to be higher than those assessed by external independent raters (Kinkaid et al., 2025). Developing methods for fidelity measurement that balance practicality with accuracy is challenging and will require consultation with services about what is feasible.

A potential limitation of the current study is our use of convenience sampling. This was considered a pragmatic choice for an initial multi-site study but risks selection bias. Services that would return lower fidelity scores may have been less likely to participate, for example, those with poorer data quality (i.e., higher rates of missing data) or those under resource constraints. Consequently, the findings may overestimate average fidelity scores over the FREED network. Future research should aim to include all FREED services. Framing future interviews to emphasise learning and development, rather than evaluation, may also encourage participation from a broader range of services and minimise bias.

Another potential development to enhance rating validity is triangulation, which involves gathering information from multiple sources to confirm scoring (Bond & Drake, 2020). For example, complementary interviews with FREED patients and their carers could ask for personal insights and commentary for each item/component score, existing items could be adapted for their ratings, or new items could be developed to evaluate what patients and their families and supporters identify as important.

## 5. Conclusions

Ongoing fidelity measurement is expected to improve model adherence over time (Williams et al., 2021). It can also develop our understanding of how well FREED adapts to local contexts and whether certain variations in delivery compromise its core, evidence-based aims and components (Ferrari et al., 2023). However, maintaining consistent, high-quality fidelity assessment across FREED services will require sustained investment and infrastructure. Similarly, additional funding is needed to allow rapid access to early intervention services across the country.

## Figures and Tables

**Table 1:** Fidelity scores, labels, and weighting.

Overall Score Label	% Score	Weighted Summed Score
Not satisfactory	<60%	<299
Lower fidelity	60–69%	300–344
Medium fidelity	70–79%	345–394
High fidelity	80–89%	395–444
Maximum fidelity	>90%	445–500

**Table 2:** Fidelity scores and referral distribution across participating FREED services (January to June 2025).

Service ^1^	Service Description	Referrals (*n*)	Overall Fidelity Score (%)	Component 1: Rapid Access Targets (%)	Component 2: Care Package and Data (%)
Whole network	-	242	72	57	77
SLaM	Adult service Urban	36	60	20	75
A	Adult service Urban/rural	52	69	63	78
B	All-age Urban/rural	68	72	45	82
C	Adult service Urban/rural	17	79	87	76
D	Adult service Urban/rural	31	70	63	72
E	Adult service Urban/rural	38	81	85	81

^1^ To preserve service confidentiality, service names outside of the original FREED service (South London and Maudsley NHS Foundation Trust [SLaM]) have been replaced with letter descriptors.

## Data Availability

The data used in this study is not publicly available.

## Note

1 Missing data are partly due to a lag in data collection (e.g., referrals may not yet have an agreed diagnosis whilst waiting for assessment); patient data are updated over time as patients progress through assessment and treatment.

## Supplementary Materials

The following supporting information can be downloaded at https://www.mdpi.com/article/10.3390/bs15111521/s1: Table S1: FREED Fidelity tool scoring sheet; Document S2: FREED Fidelity items information; Document S3: FREED Fidelity assessment interview prompts.

## Abbreviations

The following abbreviations are used in this manuscript:

NHS	National Health Service
FREED	First Episode Rapid Early Intervention for Eating Disorders
ED	Eating Disorder
DUED	Duration of Untreated Eating Disorder
RE-AIM	Reach, Effectiveness/Efficacy, Adoption, Implementation, Maintenance
KCL	King’s College London
IRR	Inter-rater Reliability
ICC	Intraclass Correlation Coefficient
COVID	Coronavirus Disease
ARFID	Avoidant/Restrictive Food Intake Disorder
EDCRN	Eating Disorders Clinical Research Network
SLaM	South London and Maudsley

## Appendix A. Inter-Rater Reliability for All Sites

**Site**	**Kappa Quadratic**	**Kappa Linear**	**ICC3k**
SLaM	0.991	0.980	0.998
A	0.983	0.949	0.992
B	0.941	0.871	0.970
C	0.933	0.859	0.966
D	0.934	0.880	0.968
E	0.967	0.915	0.984
