# Supplementary material for: Implementation Fidelity in Early Intervention for Eating Disorders—A Multisite Pilot Study
Source: Behav Sci (Basel). 2025 Nov 8;15(11):1521. doi: 10.3390/bs15111521 (PMC12649207; doi:10.3390/bs15111521)
Supplement: Supplementary file 1 [file behavsci-15-01521-s001.zip › Document Supplement S2 - Fidelity items information.pdf]

Supplement 2. Fidelity assessment item guide

Guide - Fidelity measurement in First Episode Rapid Early Intervention for Eating Disorders (FREED) services

Table of Contents

Introduction 4

FREED compared to conventional treatment for eating disorders 8

The 48-hour engagement call 10

The FREED assessment 11

Treatment on the FREED pathway 13

Referral route 15

Service availability 15

FREED Champion 16

FREED Mini team and huddle 16

Clinical supervision 17

FREED data tracker 17

Duration of untreated eating disorder (DUED) 17

Active support 18

Training 18

Transitions 18

Community awareness, education, and outreach 19

Diversity and inclusion of under-served groups 20

Introduction

This document contains more detailed explanations of the rationale behind each fidelity tool item. It should be read in combination with the fidelity tool scoring sheet.

Sixteen items in the assessment are quantitative and can be assessed using routinely collected FREED data. The remaining 19 items require assessment via a semi-structured interview with a FREED Champion.

First the core aims and functions, and principles of early intervention for eating disorders (ED) are outlined, followed by the service model and key components for a FREED-inspired early intervention service.

Then, the FREED model is outlined and compared to conventional ED treatment.

Finally, items and their rationale and associated measurement are described.

Early intervention for eating disorders

"Early intervention is the early detection of (emerging) disease, together with easy undisrupted access to care, followed by rapid provision of illness and developmental stage-specific, proportionate and personalised intervention, for as long as necessary and effective and by services that are inclusive and youth- and family-friendly and span the peak period of onset."

Core aims and functions

The specific aims and key functions of an early intervention service, guided by current international evidence and experience of delivering early intervention programmes for eating disorders.

- Early detection and referral, including for people with emerging symptoms
- Reduce duration of untreated eating disorder
- Focus on facilitating and empowering young person to make early changes
- Care is responsive to and intensified by individual need
- Evidence-based interventions focussing on full recovery, i.e., restoring optimal functioning and developmental trajectory
- Integrated care with easy transitions, appropriate to individual stage of life
- Establishing meaningful connections with family & community

Text inspired by and adapted from the Early Psychosis Prevention and Intervention Centre model (ORYGEN). Definition of early intervention, adapted from McGorry, Ratheesh, O'Donoghue (2018); JAMA Psychiatry.

Principles

The guiding principles of a service dedicated to achieving the aims and functions of early intervention for eating disorders

The service is:

- Actively raising awareness and reducing stigma
- Providing expert care that is easily accessible & responsive
- Delivering developmentally-informed care sensitive to individual characteristics
- Providing a coordinated service approach
- Providing a holistic & collaborative approach to intervention
- Carrying a pervasive spirit of optimism
- Guided by evidence in clinical practice
- Person-centred and young-person friendly
- Motivational and meets the person where they are at
- Family friendly and actively encourages involvement of close others & supports them
- Available for all diagnoses and presentations
- Collaborating and partnering with local health care providers & other community and educational organisations focused on emerging adults

Text inspired by and adapted from the Early Psychosis Prevention and Intervention Centre model (ORYGEN).

Service model and care pathway components

These components are the essential elements of a comprehensive early intervention eating disorders model and service pathway, such as First Episode Rapid Early Intervention for Eating Disorders (FREED).

- Easy access to services
- Evidence-based treatments with adaptations for emerging adulthood
- Early intervention champions and dedicated mini team
- Motivational style and psychoeducation
- Active engagement upon referral
- Focus on early changes
- Good transition management
- Family involvement, programs and family peer support
- Youth participation and peer support
- Workforce development needs
- National network for collaboration and supervision
- Clinical supervision
- Community awareness and education
- Partnerships
- Routine data collection to evaluate implementation fidelity and treatment effectiveness.

Text inspired by and adapted from the Early Psychosis Prevention and Intervention Centre model (ORYGEN).

FREED compared to conventional treatment for eating disorders

Table 1. FREED compared to conventional ED service, from Fukutomi et al. (2020)

	FREED	Conventional ED service
Prioritisation	• Based on the model of illness that emphasizes 'biological malleability' during early illness stages and hence prioritizes these cases	• Prioritisation based on diagnosis and/or severity of illness
Access	• Easy access. Encourage early referral from primary care. Working to introduce self-referral in future	• Multiple service barriers to referral; early referrals not actively encouraged
Aims and objective	• To deliver a rapid, person-centred and effective service for young people with EDs that reduces duration of untreated ED and promotes early full recovery	• To deliver best possible care to all patients seen
Approach	• Person-centred care determined by stage of illness	• One size fits all; standard packages of care determined by diagnosis or severity
Care model	• Evidence-based treatments, developmentally tailored and appropriate for stage of illness	• Either child and adolescent-centred or adult centred approach with either predominantly family-based or individual treatment approaches
Engagement	• Active outreach to patient (e.g. 48-hour call)<br>• Multiple modes of contact (e.g., emails, text and call)<br>• Flexibility (e.g., changing timetable and, cancellations)<br>• Information resources tailored to young people (e.g., information on role of social media in maintenance of ED symptoms and advice on responsible use of social media)	• Onus on patient to contact service (e.g., opt in letter)<br>• Traditional forms of contact (e.g., letters)<br>• Administration team as initial point of contact<br>• Limited flexibility reappointments
Assessment	• Biopsychosocial and person centred, including focus on young person's needs, priorities and strengths<br>• Actively encourage family attendance	• Variable family involvement<br>• Limited psychoeducation

	• Psychoeducation emphasizing ‘biological malleability’<br>• Explore social media use	
Treatment	• Tailored evidence-based treatments<br>• Focus on early nutritional change<br>• Family education, skills training and support<br>• Use of technology (online interventions, using phones and apps)<br>• Focus on transition management (e.g. university starter groups, close liaison with child and adolescent ED service)	• Variable focus on nutritional change<br>• Variable family involvement<br>• Variable use of technology<br>• Some issues with transitions of care

The 48-hour engagement call

Source: (Engagement protocol for FREED patients; Champion Pack)

The young person should be called by a clinician within 48-hour of the referral being received within the service. There are four key aims of this call:

1. To engage the patient
2. To undertake a brief screen for onset of eating disorder (and where possible ascertain suitability for FREED)
3. Provide key information about the service and about FREED
4. When appropriate, book the assessment appointment.

Collecting data on this item is important to see if FREED patients are being engaged in a consistent and timely fashion, and to see how early change is fostered in both your early intervention service, and nationally.

How this is measured in the fidelity assessment:

First call attempt date (Item 1)

Actual call date (Item 2)

Data availability (Item 3, 4)

How to score/ provide evidence for these items in the fidelity assessment:
These are scored via FREED tracker data (the engagement call attempt and actual date columns).

The FREED assessment

Source: FREED training day slides, FREED assessment cover sheet

FREED aims to take an optimistic, motivational, collaborative, change-focused stance from assessment onwards. The goal is for a FREED assessment to take place within 2 weeks of being referred into the service. It's important to try and meet this target to avoid disruption to the young person's life caused by the eating disorder and avoid the possibility of deteriorating on waiting lists without any active input.

The following items (or as much as possible) should be discussed with the patient in the assessment, with a motivational interviewing stance – explore existing knowledge, ask permission to share new information, use diagrams and different information formats:

- Rationale for early intervention incl. biological malleability of brain changes & the importance of first 3 years.
- The impact of ED on brain, body & behaviour.
- Early dietary & symptom change.
- Exploration of social media use.
- Exploration of recent or pending transitions (e.g. out of school; to university; to work).
- Exploration of identity & emerging adulthood.
- Carer involvement & support from others.

We try to help FREED patients set initial goals at the end of their assessment; this should include an initial nutritional care plan. As per the FREED assessment cover sheet, the following psychoeducation materials should be provided, if appropriate:

- FREED specific psychoeducation
- The brain and eating disorders
- Social media and apps
- Preparing for university
- Other psychoeducation

Appropriate referrals should also be made, for example, to:

- Carer support
- Dietician
- Family therapy
- Intensive treatment
- PEACE pathway
- Occupational Therapy
- University preparation group

How this is measured in the fidelity assessment:

Assessment appointment offered within 2 weeks (Item 5)

Actual time to assessment (Item 6)

Data availability (Item 7, 8)

Motivational stance (Item 14)

Adaptations to assessment (Item 29)

Involvement of family and close others (Item 31)

How to score/ provide evidence for these items in the fidelity assessment:

Items 5-8 are scored via FREED tracker data (Assessment offered and actual date columns). For assessment style, this can be scored via interview. You can also provide evidence of use of FREED assessment cover sheets (anonymised), patient testimonials, family testimonials, service protocol developments etc.

Treatment on the FREED pathway

Source: FREED Champion pack, Determining start of treatment

Treatments are developmentally adapted to be relevant for the FREED population. Many services are implementing new, shorter, evidenced-based treatments including guided self-help. We suggest you think about these treatments and how to 'FREEDify' them. For example, the FREED treatment package may have a small increase in number of outpatient sessions offered so that, in addition to individual treatment, FREED patients may also have access to family sessions, workshops, dietetics etc.

Adaptations to treatment in FREED include:

- Tailored evidence-based treatments.
- Focus on early nutritional change.
- Family education, skills training and support.
- Use of technology (on-line interventions, using phones, apps).
- Focus on transition management (e.g., university starter groups, close liaison with Child and Adolescent Eating Disorder services (CAEDS)).

There are two key time points that are of interest in FREED:

1. The start of any type of active, change-focused support; and
2. The start of evidence-based treatment.

Here, we are measuring the latter - evidence-based treatments, i.e., those within the National Institute for Health and Care Excellence (NICE) guidelines. The recording of other brief interventions or 'active support' sessions are detailed on the Active support page. See the 'Determining start of treatment' resource for further information on how to calculate this and what treatments are included as part of this. Determining start of evidence-based treatment is important for calculating duration of untreated eating disorder (DUED) and other process variables such as waiting times.

The goal is for evidence-based treatment to take place within 4 weeks of being referred into the service. This is because we know that longer waiting times are linked to worsening symptoms, poorer outcomes, and higher mortality for those with eating disorders (Carter et al., 2012; Solmi et al., 2024). It's important to collect data on treatment waiting times as currently, within adult ED services in England, very few evaluations of adherence to waiting time targets have been conducted (Richards et al., 2021; Ayton et al., 2022).

How this is measured in the fidelity assessment:

Treatment start within 4 weeks (Item 9)

Actual time to start treatment (Item 10)

Data availability on treatment wait times (Item 11, 12)

Treatments offered are evidence-based (Item 15)

Proportion receiving evidence-based treatment (Item 16)

Treatment style (Item 30)

Involvement of family and close others (Item 31)

How to score/ provide evidence for these items in the fidelity assessment:

Items 9-16 can be scored via FREED tracker data (Treatment offered and actual date, and treatment type columns). Items 30 and 31 can be scored via interview. Patient and family testimonials or submissions and protocol documents can also contribute to Items 30 and 31.

Relevant external resources:

NICE guidelines for eating disorder treatment

Referral route

There are currently multiple ways a young person can enter a specialist eating disorder service. Self-referral for young people aged under 18 in the UK is allowed and encouraged, but this option is not yet required for those aged 18 and over, adding an extra obstacle to early intervention for young adults (Mills et al., 2023). An expert consensus consortium of clinical and academic researchers recommended all-age self-referrals, to facilitate direct access to early intervention and specialist care (Davey et al., 2023). Access to early intervention and FREED should be as easy as possible for a young person. This includes encouraging early referrals from primary care but ideally, work should be in place for self-referral to be possible.

How this is measured in the fidelity assessment:

Referral route (Item 13)

How to score/ provide evidence for these items in the fidelity assessment:

Via interview.

Service availability

FREED is designed as a transdiagnostic service for emerging adults with any eating disorder, regardless of DSM-5 severity indicators, and across the 16–25-year age range. As such, a FREED service operating with maximum fidelity would not restrict access to the service based on age, diagnosis or eating disorder severity level. These restrictions in care are often determined by commissioning, or where services are split into separate child and adolescent eating disorder services (CAEDS) and adult eating disorder services (AEDS). Accordingly, scores here will consider different approaches to these restrictions, such as incidences where care is outsourced to a third sector organisation based on diagnosis or severity criteria.

Note. The fifth edition of the Diagnostic and Statistical Manual for Mental Disorders classifies severity of anorexia nervosa by body mass index (BMI; kg/m2); for bulimia nervosa, severity is measured by frequency of inappropriate compensatory behaviours (e.g., vomiting), and for binge eating disorder, severity is measured by frequency of binge eating episodes.

How this is measured in the fidelity assessment:

Diagnosis, ED presentation, and age items (17-19)

How to score/ provide evidence for these items in the fidelity assessment:

The FREED tracker should contain the majority of information needed to score these items, if it is sufficiently completed. For ED presentation item (Item 17, 19), this can be assessed at interview. Any additional information can be uploaded as a supplement (e.g., documents or written information about referrals to other organisations for 'milder' presentations or outsourcing of treatment).

FREED Champion

FREED Champions are crucial to the success and embedding of FREED in the service and ensuring the model is being upheld in the service. The FREED champion should be a clinician of any background (psychologist, nurse therapist, psychotherapist), typically at Band 7 level and 0.6 whole time equivalent (WTE) per week, due to the managing aspect and range of clinical decisions that need to be made within this role.

How this is measured in the fidelity assessment:

FREED Champion – post and WTE (Item 20, 21)

How to score/ provide evidence for these items in the fidelity assessment:

Interview that confirms the role and WTE for the FREED Champion.

FREED Mini team and huddle

Source: FREED implementation guide, Huddle Agenda Template

In most services, there should be a 'FREED mini team' who work with the FREED champion to provide assessment and treatment for FREED patients. The size of this team will depend on the size of the service and the number of FREED referrals, but is likely to vary from 2 clinicians for a small service up to 10 for a large one. Services have found having a weekly, 15 minute 'FREED huddle' helps to ensure that FREED processes are being followed. The FREED Champion and mini team should also make an effort to feedback to the wider service and in business meetings about FREED to ensure that FREED is kept 'on the radar' for the whole service.

The huddle agenda should cover the following:

- Current wait for assessment and treatment
- Patients needing allocation
- Vacant therapy slots
- Assessments, DNA's, & cancellations
- Patients in treatment who may be disengaging
- New treatment cases
- Changes in treatment status (e.g., stepping up)
- CAMHS transitions

How this is measured in the fidelity assessment:

FREED mini team and FREED huddle (Item 22, 23)

How to score/ provide evidence for these items in the fidelity assessment:

Via interview. Example huddle minutes can also be uploaded as a supplement.

Clinical supervision

Source: FREED Champion pack

Clinical supervision is an essential place for the FREED team to discuss cases, ensure that treatments are matching an individual's life stage, and check that treatment provided continues to stay 'on model'.

How this is measured in the fidelity assessment:

Clinical supervision (Item 24)

How to score/ provide evidence for these items in the fidelity assessment:

Via interview.

FREED data tracker

Source: FREED Champion pack

The tracker is a major component of the FREED Champion role and an integral part of keeping FREED an evidence-based intervention. Without data, it's difficult to showcase how many patients are benefitting from FREED and to ultimately show that early intervention works. It is important to show that the FREED data tracker is submitted regularly with sufficient completion of most items (including pre- and post-treatment outcome measures).

How this is measured in the fidelity assessment:

Tracker (Item 25)

How to score/ provide evidence for these items in the fidelity assessment:

This will be assessed based on how frequently the FREED tracker is submitted, in addition to whether the mandatory data collection items (columns highlighted in blue) are routinely collected.

Duration of untreated eating disorder (DUED)

DUED is the time between clinical onset of an eating disorder and first contact with specialist eating disorder services. Calculating DUED is important within FREED as it helps to determine eligibility for FREED. It is also an important research concept and key outcome for early intervention, with data on DUED being reported in most FREED publications (e.g., Flynn et al., 2021).

How this is measured in the fidelity assessment:

DUED information collection (Item 26)

How to score/ provide evidence for these items in the fidelity assessment:

This will be scored based on how complete DUED data collection is in the FREED tracker.

Active support

Source: Determining start of treatment

Ideally, patients would progress straight from assessment to evidence-based NICE-concordant treatment. However, many services are stretched and even in FREED, there can be waits for treatment. We know services are working hard to bridge this period by helping patients take first steps towards recovery, and we want to capture this support. Start of active support is the date at which the patient first starts any type of active, change- and recovery-goal focused intervention (including evidence-based treatment).

Active support includes any change-focused activities whether delivered via psychoeducational workshops and groups, one-to-one support sessions, peer support, dietetic sessions/support, social interventions, brief interventions, and review meetings with psychoeducation and/or other change-focused activities. These activities will often occur less than weekly but would be expected to happen at least monthly. The following activities are not considered as the start of active support: solely monitoring, checking-in or "holding" someone.

How this is measured in the fidelity assessment:

Active support (Item 27)

How to score/ provide evidence for these items in the fidelity assessment:

This item can be measured via completeness in the FREED tracker ('active support' columns, to measure availability of active support for FREED patients).

Training

Having completed the FREED online training package is essential for the FREED Champion and mini team. It is also important that the FREED Champion has attended the live training day organised by the FREED national team. It is important that services also keep engaged with new training relevant to early intervention and invest in workforce development (e.g., training in evidence-based treatments)

How this is measured in the fidelity assessment:

Training (Item 28)

How to score/ provide evidence for these items in the fidelity assessment:

Via interview.

Transitions

Source: Implementation guide, transitions comms pack

Age-related service transitions between child and adolescent and adult eating disorders services present significant challenges and opportunities for service users

and staff. Making a fresh start allows a fresh look at a person's difficulties and may unlock new approaches to treatment and care. However, poor communication and coordination between services and other issues contribute to such transitions also being associated with distress, treatment delays, drop-out and deterioration.

In FREED, a great emphasis is placed on giving attention to the different transitions that arise in a young person's life (such as transitions out of child/adolescent services and those to university, employment and other adult roles). This is a key part of the FREED care package. We have specific training and resources available to support professionals to deliver better age-related eating disorders service transitions (https://freedfromed.co.uk/eating-disorders-service-transitions).

The Royal College of Psychiatrists detail a list of key recommendations for better transitions (see college report CR208):

- Awareness of the problem
- Early identification and notification of the need for transition
- Involvement of family and carers
- Flexible timing of transition
- Close links between services
- Transition coordinator
- Provide good information
- Clear protocols and pathways
- Patient-centred transition plan
- Multidisciplinary discharge planning meeting
- Overlap period of joint working
- Respect for attachments and therapeutic alliances

How this is measured in the fidelity assessment:

Age-related transitions (Item 32), University transitions (Item 33)

How to score/ provide evidence for these items in the fidelity assessment:

This can be scored via interview. Patient testimonials may also be provided to explain the transition process and experience within the service.

Relevant external resources: RCPSYCH College Report CR208

Community awareness, education, and outreach

Source: FREED Champion pack

Community awareness and education:

Outreach: Working with GPs and spreading the word that FREED is available is an often challenging but important part of the FREED Champion's role, given that GPs don't have a lot of time, and there remains stigma and a lack of understanding about eating disorders. Our top tips include reaching out to those GPs who you and your service have good relationships with and ask them how might be best to spread the

word of FREED. A GP FREED guide and video are available to help get started with this.

How this is measured in the fidelity assessment:

Community awareness and education, outreach (Item 34)

How to score/ provide evidence for these items in the fidelity assessment:

Via interview.

Diversity and inclusion of under-served groups

Source: Equality and Diversity in FREED training module

In FREED, we have to make extra efforts to reach and care for certain populations to ensure that everyone can access and benefit from early intervention. Under-represented and under-served groups include racial, ethnic, and/or cultural minorities, those from a lower socioeconomic status or background, higher-weight or overweight individuals, men, and LGBTQ+ individuals (lesbian, gay, bisexual, transgender, queer or questioning, intersex, asexual, and more). Other potentially under-served groups include those with physical disabilities or health problems, autistic people and those with attention-deficit/hyperactivity disorder (ADHD), and those with complex presentations.

Extra considerations should be made when thinking about family involvement, emerging adulthood experiences, and transitions for these individuals. Focus groups and interviews are excellent ways to understand the experiences and views of under-represented individuals and groups. More targeted outreach may then be needed after establishing gaps in care. It may also be a good idea to monitor your service's diversity tracker data at a local level, and to compare this to wider service and national baseline rates (e.g., from ons.gov.uk).

There are also important aspects of the service environment that need to be considered to promote diversity and inclusivity. For example, in recruitment processes and staff representation. The service environment should also be accommodating and safe for all people, such as ensuring chairs accommodate for all weights, having leaflets and resources in multiple languages, and ensuring your website shows a variety of people, e.g., different genders, varied body sizes, and ethnicities.

How this is measured in the fidelity assessment:

Diversity and inclusion of under-served groups (Item 35)

How to score/ provide evidence for these items in the fidelity assessment:

Via interview. Patient testimonials can be provided as supplement to this.
