# Supplementary material for: Implementation Fidelity in Early Intervention for Eating Disorders—A Multisite Pilot Study
Source: Behav Sci (Basel). 2025 Nov 8;15(11):1521. doi: 10.3390/bs15111521 (PMC12649207; doi:10.3390/bs15111521)
Supplement: Supplementary file 1 [file behavsci-15-01521-s001.zip › Document Supplement S3 - Fidelity assessment interview prompts.pdf]

Supplement S1. Fidelity assessment interview prompts.

FREED Fidelity Assessment

The aim of this assessment is to investigate whether your service covers the core functions, principles, and components of an early intervention pathway for emerging adults with eating disorders, specifically, the operation of a FREED pathway tailored to recent onset eating disorders across ages 16-25.

1. Item 13: How are potential FREED patients referred into your service?

How much time does it take to process referrals? Are there any delays – why & how often?

2. Item 17: Do you accept all eating disorder presentations onto the FREED pathway (all diagnoses and irrespective of 'severity' levels)? (see notes below)

Note: Diagnoses seen on the FREED pathway include anorexia and bulimia nervosa, atypical anorexia and bulimia nervosa, binge eating disorder, avoidant restrictive food intake disorder (ARFID), and other specific feeding or eating disorder (OSFED). Severity indicators sometimes change what care is offered to certain patients. In the DSM-5, these are as follows; Anorexia nervosa level of severity: Mild: BMI > 17 kg/m2; Moderate: BMI 16-16.99 kg/m2; Severe: BMI 15-15.99 kg/m2; Extreme: BMI < 15 kg/m2. Bulimia nervosa level of severity: Mild: An average of 1-3 episodes of inappropriate compensatory behaviours per week; Moderate: An average of 4 -7 episodes of inappropriate compensatory behaviours per week; Severe: An average of 8 -13 episodes of inappropriate compensatory behaviours per week; Extreme: An average of 14 or more episodes of inappropriate compensatory behaviours per week. Binge eating disorder level of severity: Mild: 1-3 binge-eating episodes per week; Moderate: 4-7 binge-eating episodes per week; Severe: 8-13 binge-eating episodes per week; Extreme: 14 or more binge-eating episodes per week.

3. Item 19: Do you see all ages intended within the FREED pathway (16-25, or 18-25 if adult service)?

4. Item 20: Do you have a FREED Champion? How is the FREED Champion post coordinated?

What professional background and grade are they? How are they supported? How well does this work?

5. **Item 21: How much WTE is the FREED Champion assigned to work on FREED & for what catchment area?**

It's suggested the Champion role is 0.6 WTE per week or 3 days per ~2 million catchment area? 0.2 FTE or 1 day of this time is suggested for champion-specific duties, 0.4 FTE or 2 days is suggested for extra time for FREED patients.

Do you think this is appropriate for the catchment area?

Is Champion time protected?

6. **Item 22: Is there a dedicated FREED mini team?**

Do the team work well together? Is their time protected for FREED?

7. **Item 23: Is there a weekly FREED huddle meeting?**

How well does this work?

8. **Item 24: Are there regular specific clinical supervision meetings for FREED?**

How well does this work?

9. **Item 14: If a patient on the FREED pathway is ambivalent about treatment, how is this approached? How do the FREED team work to increase motivation to change?**

Are the FREED Champion/mini team aware of motivational training techniques? Have they had any training? What was the nature and intensity of this training? Do you use these techniques regularly?

10. **Item 15: What treatments do you offer to FREED patients?**

Do all patients receive 'evidence based' treatments? Is there a selection of treatments tailored to patients' needs?

Note. NICE guidelines advise eating disorder-focused Cognitive Behaviour Therapy (CBT-ED), Maudsley Anorexia Nervosa Treatment for Adults (MANTRA), Specialist Supportive Clinical Management (SSCM) or eating disorder-focused Focal Psychodynamic Therapy (FPT) for adults with anorexia nervosa and similar presentations; anorexia nervosa-focused family therapy (FT) for children and young people with anorexia nervosa; Cognitive Behaviour Therapy guided self-help or group CBT-ED or individual CBT-ED for adults with bulimia nervosa, binge eating disorder and similar presentations; and bulimia nervosa-focused family therapy (FT) for children and young people with bulimia nervosa.

11. **Item 27: Do you offer any kind of waitlist interventions or support whilst people are waiting for evidence-based treatment?**

12. Item 28: Who has completed the online FREED training?

13. Item 29: Can you describe how the FREED assessment is conducted? How is this different to other assessments?

What topics are usually discussed at a FREED assessment?

14. Item 30: How is treatment adapted to meet the needs of FREED patients?

For example, how is early change encouraged? Is dietetic input available?

15. Item 31: How do you encourage young people to involve family members or close others in assessment and treatment in FREED?

At what points during pathway? (call, assessment, treatment
What happens if the young person is ambivalent about involving close others or refuses outright to involve them?
Do you offer interventions tailored to family involved (e.g., FT-AN for emerging adults)
Do you do any parent/carer groups?

16. Item 33: How are age-related service transitions managed in your service?

17. Item 34: How do you prepare for or acknowledge transitions to university?

18. Item 34: Do the FREED team engage in any community awareness and education activities around FREED, eating disorders, and/or early intervention? e.g. outreach activities to GPs, schools, etc.?

19. Item 35: What initiatives are in place to promote equality and diversity in the service (see notes below)

What about within FREED?

Note. Under-served communities in eating disorders include men with eating disorders, black and minority ethnic groups, and those who identify as LGBTQ+, those at a higher weight, neurodivergent

people and people with learning disabilities. Inclusivity in the service and diversity may look like the following initiatives/changes:

- *Improved diverse practices in recruitment, and more representative staffing*
- *Adapted, enhanced, or specific treatment pathways to accommodate for specific additional needs (e.g., PEACE pathway for autistic people)*
- *Ensuring service environment accommodates for all people (e.g., modifying the built environment – chairs to accommodate for all weights)*
- *Ensuring diversity in service resources (e.g., website shows people of varied body sizes and/or ethnicities, leaflets in multiple languages)*
