# Supplementary material for: Implementation Fidelity in Early Intervention for Eating Disorders—A Multisite Pilot Study
Source: Behav Sci (Basel). 2025 Nov 8;15(11):1521. doi: 10.3390/bs15111521 (PMC12649207; doi:10.3390/bs15111521)
Supplement: Supplementary file 1 [file behavsci-15-01521-s001.zip › Table S1. FREED Fidelity tool scoring sheet.pdf]

**Supplement S1. Fidelity tool scoring sheet**

| Item # | Criterion | How assessed | Scoring | | Weight | Score |
|---|---|---|---|---|---|---|
| 1 | **Engagement call date**<br>***Attempted*** within 48 hours of referral | From FREED tracker<br><br>for whom data are available (% within completed cells) | Not satisfactory: 1 | <60% receive call attempt within 2 days | 6.82% | |
| | | | Lower fidelity: 2 | 60-69% receive call attempt within 2 days | | |
| | | | Medium fidelity: 3 | 70-79% receive call attempt within 2 days | | |
| | | | High fidelity: 4 | 80-89% receive call attempt within 2 days | | |
| | | | Maximum fidelity: 5 | ≥90% receive call attempt within 2 days | | |
| 2 | ***Actual*** call date | From FREED tracker<br><br>for whom data are available (% within completed cells) | Not satisfactory: 1 | <60% receive call within 2 days | 2.27% | |
| | | | Lower fidelity: 2 | 60-69 receive call within 2 days | | |
| | | | Medium fidelity: 3 | 70-79% receive call within 2 days | | |
| | | | High fidelity: 4 | 80-89% receive call within 2 days | | |
| | | | Maximum fidelity: 5 | ≥90% receive call within 2 days | | |

| Item # | Criterion | How assessed | Scoring | | Weight | Score |
|---|---|---|---|---|---|---|
| 3<br><br>4 | **Data availability: Percentage** of cases for whom engagement call data is **available** | FREED tracker total missing percentage for **offered** call (Item 3) and **actual** call (Item 4) | Not satisfactory: 1<br>Lower fidelity: 2<br>Medium fidelity: 3<br>High fidelity: 4<br>Maximum fidelity: 5 | <60% data available<br>60-69% data available<br>70-79% data available<br>80-89% data available<br>>90% data available | 2.27%<br><br>2.27% | Offered:<br><br>Actual: |
| 5 | **Assessment appointment offered within 2 weeks** | From FREED tracker, for whom data are available (% within completed cells) | Not satisfactory: 1<br>Lower fidelity: 2<br>Medium fidelity: 3<br>High fidelity: 4<br>Maximum fidelity: 5 | <60% seen within 2 weeks<br>60-69 seen within 2 weeks<br>70-79% seen within 2 weeks<br>80-89% seen within 2 weeks<br>>90% seen within 2 weeks | 6.82% | |
| 6 | **Actual time to assessment** | Tracker, for whom data are available (% within completed cells) | Not satisfactory: 1<br>Lower fidelity: 2<br>Medium fidelity: 3<br>High fidelity: 4<br>Maximum fidelity: 5 | <60% seen within 2 weeks<br>60-69 seen within 2 weeks<br>70-79% seen within 2 weeks<br>80-89% seen within 2 weeks<br>>90% seen within 2 weeks | 2.27% | |

| Item # | Criterion | How assessed | Scoring | | Weight | Score |
|---|---|---|---|---|---|---|
| 7<br>8 | **Proportion** of people for whom assessment info is **available** | FREED tracker total missing percentage for **offered** (Item 7) and **actual** (Item 8) assessment for patients who received an engagement call | Not satisfactory: 1 | <60% data available | 2.27% | Offered: |
| | | | Lower fidelity: 2 | 60-69% data available | | |
| | | | Medium fidelity: 3 | 70-79% data available | | |
| | | | High fidelity: 4 | 80-89% data available | 2.27% | Actual: |
| | | | Maximum fidelity: 5 | ≥90% data available | | |
| **9** | **Treatment starts within 4 weeks** Proportion of people for whom treatment start is *offered* within 4 weeks of referral | FREED Tracker, for whom data are available (% within completed cells) | Not satisfactory: 1 | <60% offered within 4 weeks | 6.82% | |
| | | | Lower fidelity: 2 | 60-69 offered within 4 weeks | | |
| | | | Medium fidelity: 3 | 70-79% offered within 4 weeks | | |
| | | | High fidelity: 4 | 80-89% offered within 4 weeks | | |
| | | | Maximum fidelity: 5 | >90% offered within 4 weeks | | |
| 10 | **Actual time to start treatment** | FREED tracker, for whom data are available (% within completed cells) | Not satisfactory: 1 | <60% start within 4 weeks | 2.27% | |
| | | | Lower fidelity: 2 | 60-69 start within 4 weeks | | |
| | | | Medium fidelity: 3 | 70-79% start within 4 weeks | | |
| | | | High fidelity: 4 | 80-89% start within 4 weeks | | |
| | | | Maximum fidelity: 5 | >90% start within 4 weeks | | |

| Item # | Criterion | How assessed | Scoring | | Weight | Score |
|---|---|---|---|---|---|---|
| 11<br>12 | **Proportion** of people with available data. | FREED tracker total missing percentage for **offered** (Item 11) and **actual** (Item 12) treatment start for patients who have an assessment date | Not satisfactory: 1 | <60% data available | 2.27%<br><br>2.27% | Offered: |
| | | | Lower fidelity: 2 | 60-69% data available | | |
| | | | Medium fidelity: 3 | 70-79% data available | | Actual: |
| | | | High fidelity: 4 | 80-89% data available | | |
| | | | Maximum fidelity: 5 | ≥90% data available | | |

## Service model and care package

| Item # | Criterion | How assessed | Scoring | Weight | Score |
|---|---|---|---|---|---|
| 13 | **Referral route** | Interview | • (1) Via gatekeeping processes, lots of delays, more than two weeks between referral and approval, patients occasionally get lost in the system, dropping out.<br>• (2) Some gatekeeping processes. It typically takes a week or more between referral and approval. Potential for patients to get lost in the system but not frequent.<br>• (3) A GP referral is sent to a Single Point of Access, before being forwarded to the ED service. Small delays but processed within a few days/less than a week.<br>• (4) A GP or Single Point of Access referral direct to ED service –no delays apart from exceptional cases<br>• (5) Via self-referral | 2.27% | |
| 14 | **Motivational stance** of FREED clinicians | Interview | • (5) Whole FREED team (or key people) have received motivational interviewing training and use these techniques regularly<br>• (4) Champion has had motivational interviewing training, and/or answer demonstrates that these kinds of techniques are routinely used, or team work hard to increase motivation even if no training day<br>• (3) Motivational interviewing resources mentioned and regularly used but no training, evidence that these techniques are used regularly. | 2.27% | |

| Item # | Criterion | How assessed | Scoring | Weight | Score |
|---|---|---|---|---|---|
| | | | • (2) Some procedures mentioned. Techniques to increase motivation appear limited or not used regularly.<br>• (1) Brief answer given with no mention of motivational interviewing techniques, team conversations, or approach taken to tackle motivational difficulties. No training in motivational interviewing. | | |
| 15 | **Treatments offered are within the NICE-guidelines recommended treatments for EDs** | Interview & FREED tracker ("Tx type" column) as supplementary | • (1) Monitoring and general support is provided **without** evidence-based psychological treatments.<br>• (2) Monitoring and general support is provided with **limited use** of evidence-based psychological treatments.<br>• (3) Some evidence-based treatments are offered, but not equitably.<br>• (4) All patients receive evidence-based care, however, a limited number of treatments are available (e.g., group CBT-ED but not individual CBT-ED, or for AN, CBT-ED but not MANTRA or SSCM).<br>• (5) All ED patients receive evidence-based care and a range of NICE-recommended therapies are offered. Where treatment deviates from first-line NICE recommendations, this is because the patient has already received evidence-based ED treatment and consideration is being given to evidence-based treatment for | 4.55% | |

| Item # | Criterion | How assessed | Scoring | | Weight | Score |
|---|---|---|---|---|---|---|
| | | | comorbid difficulties presenting alongside the ED. | | | |
| 16 | **Proportion** of people who receive evidence-based treatment.<br><br>For those with data in treatment column, x% receive evidence-based, NICE guidelines concordant treatment. | FREED tracker completeness (%) | Not satisfactory: 1 | <60% receive or no data available | 2.27% | |
| | | | Lower fidelity: 2 | 60-69% receive | | |
| | | | Medium fidelity: 3 | 70-79% receive | | |
| | | | High fidelity: 4 | 80-89% receive | | |
| | | | Maximum fidelity: 5 | ≥90% receive | | |
| 17 | **Eating disorder presentations accepted**<br><br>**Seeing all ED diagnoses:** *AN, Atypical AN, BN, Atypical BN, BED, ARFID, OSFED etc.* **& all severity levels seen in service:** See fidelity items explanations document | Interview<br>&<br>FREED tracker (“diagnosis” column) as supplementary | • (1) We do not see all presentations and also restrict based on severity criteria<br>• (2) Limited gatekeeping procedures e.g., only ARFID is not seen, but all weight AN/BN etc. seen.<br>• (3) There is outsourcing of certain diagnoses or ‘milder’ severity EDs – we do not stay in touch with these patients.<br>• (4) Some patients go to another service and we stay in touch, keep track of them and collect data, and have good relationships with the provider organisation.<br>• (5) All diagnoses and presentations are accepted and treated on the FREED pathway. | | 2.27% | |

| Item # | Criterion | How assessed | Scoring | | Weight | Score |
|---|---|---|---|---|---|---|
| 18 | **Proportion** of people for whom diagnosis info is **available** | FREED tracker, completeness of diagnosis column: % missingness<br>For those with an <u>actual</u> assessment date | Not satisfactory: 1<br>Lower fidelity: 2<br>Medium fidelity: 3<br>High fidelity: 4<br>Maximum fidelity: 5 | <60% data available<br>60-69% data available<br>70-79% data available<br>80-89% data available<br>≥90% data available | 2.27% | |
| **19** | **Ages seen within pathway**<br><br>16-25, or 18-25 if adult service | Interview | • (1) FREED is only rolled out for a subset of the intended age range.<br>• (5) Yes, we see all ages in FREED. | | 2.27% | |
| 20 | **FREED champion(s)**<br>Suitable post | Interview | • (1) No named FREED Champion. Champion responsibilities (e.g. screening of referrals) may be shared by members of the team/ with admin).<br>• (2) Band 6 clinician acting as FREED Champion without close senior supervision.<br>• (3) Band 6 clinician acting as FREED Champion (limited support) or really unsupported band 7<br>• (4) A named band 7 level or equivalent clinician of any background is operating the pathway, or is a really well supported band 6 | | 4.55% | |

| Item # | Criterion | How assessed | Scoring | Weight | Score |
|---|---|---|---|---|---|
| | | | • (5) The named champion has extra support from one or more staff members. | | |
| 21 | **FREED Champion has protected time** - 0/6 WTE as guide. | Interview | • (1) < 0.6 WTE per week or WTE but for really wide catchment area, or Champion time is not protected<br>• (5) > 0.6 WTE per week for appropriate catchment area | 2.27% | |
| **22** | **FREED Mini team**<br>Dedicated FREED mini team with capacity for FREED | Interview | • (5) Yes, we have a mini team that work well together and stay involved and helpful with FREED/whole team involvement<br>• (4) Yes, a team is identified and work together well, but they have other competing priorities that affect their ability to help<br>• (3) Yes, a team is identified and work together well, but only 1 or 2 people or they have other competing priorities that affect their ability to help<br>• (2) Yes, a team is identified but it doesn't work very well e.g., met and worked together post-launch but support has trailed off.<br>• (1) No team. | 2.27% | |
| **23** | **Weekly FREED huddle**<br>Weekly meeting | Interview | • (5) Yes – working really well consistently. | 2.27% | |

| Item # | Criterion | How assessed | Scoring | | Weight | Score |
|---|---|---|---|---|---|---|
| | | | • (4)<br>• (3) Yes – but perhaps with inconsistencies with how well this works.<br>• (2)<br>• (1) No. | | | |
| **24** | **Supervision**<br>**Regular specific supervision meeting for FREED (weekly/monthly)** | Interview | • (5) Yes (weekly or monthly). These work well and are productive and supportive.<br>• (4)<br>• (3) Yes, but perhaps with inconsistencies in how well these work.<br>• (2)<br>• (1) No. | | 2.27% | |
| **25** | **Tracker** | Sending data on FREED referrals each quarter | • (5) FREED tracker regularly submitted to KCL team<br>• (1) Not collecting data on FREED/Not regularly submitting tracker to KCL team | | 2.27% | |
| **26** | **Is DUED (duration of untreated ED) information collected?** | FREED tracker % missingness<br>For those with an <u>actual</u> assessment date | Not satisfactory: 1<br><br>Lower fidelity: 2<br><br>Medium fidelity: 3<br><br>High fidelity: 4<br><br>Maximum fidelity: 5 | <60% data available<br><br>60-69% data available<br>70-79% data available<br>80-89% data available<br>≥90% data available | 4.55% | |
| **27** | **Active support** | Interview | • (5) Patients start evidence-based treatment without a waitlist. | | 2.27% | |

| Item # | Criterion | How assessed | Scoring | Weight | Score |
|---|---|---|---|---|---|
| | ***Offered*** for FREED patients ahead of evidence-based treatment starting, for whom data is available | Supplementary: FREED tracker (% within completed cells)<br><br>(only count for those who have had an assessment date and have not been marked 'did not attend', and do not count cells that record evidence-based treatment as active support) | • (4) There is a waitlist for treatment >4 weeks, multiple forms of waitlist interventions are offered (e.g., group sessions, psychoeducational workshops, tailored to patient). And, this support is more than one session in length.<br>• (3) There is a waitlist for treatment >4 weeks, and one session only of some form of intervention is offered<br>• (2) There is a waitlist for treatment >4 weeks, and only medical monitoring is offered or only one resource given without change-focussed input from therapist<br>• (1) There is a waitlist for treatment >4 weeks, and no offer of any form of active, change focussed intervention/ activity. | | |
| 28 | **Training**<br><br>Who has completed the online FREED training | Interview/written submission | • The FREED Champion only = 1<br>• The FREED Champion and the mini team = 5 | 2.27% | |
| 29 | **Adaptations to assessment**<br><br>See FREED fidelity item explanation document 'The FREED assessment' – all bullet pointed items will have been met plus own-service initiatives to achieve high score. | Interview | • (5) Exceptional evidence/answer that demonstrates awareness of all components, topics discussed are tailored to individual, mention of most psychoeducational materials provided.<br>• (4) Evidence that most components are discussed/aware of and some evidence that these are tailored to individual needs. | 2.27% | |

| Item # | Criterion | How assessed | Scoring | Weight | Score |
|---|---|---|---|---|---|
| | | | • (3) Many points mentioned, no mention of tailoring this to the individual<br>• (2) Only a few points mentioned, not always relevant to developmental/illness stage<br>• (1) No evidence that assessment is different to a normal assessment | | |
| 30 | **Treatment style**<br><br>See FREED fidelity item explanation document 'Treatment on the FREED pathway' – all bullet pointed items will have been met plus own-service initiatives to achieve high score. | Interview | • (5) Exceptional evidence/answer that demonstrates awareness of all components, topics discussed are tailored to individual, mention of most psychoeducational materials provided.<br>• (4) Evidence that most components are discussed/aware of and some evidence that these are tailored to individual needs.<br>• (3) Many points mentioned, no mention of tailoring this to the individual.<br>• (2) Only a few points mentioned, not always relevant to developmental/illness stage.<br>• (1) No evidence that treatment is different to normal. | 2.27% | |
| 31 | **Involvement of family and close others** | Interview | • (5) We always actively encourage young people to involve family members and/or significant others from engagement call onwards. I the person is ambivalent about or initially declines involvement we explore reasons for this. Depending on what these reasons are we will revisit the topic at intervals. In addition, the service makes use of developmentally tailored interventions such as FT- | 2.27% | |

| Item # | Criterion | How assessed | Scoring | Weight | Score |
|---|---|---|---|---|---|
| | | | AN for emerging adults and holds parent/carer groups/sessions.<br>• (4) We always actively encourage young people to involve family members and/or significant others at assessment and start of treatment. This is frequently revisited if the person initially declines involvement. And, we hold parent/carer groups/sessions in the service.<br>• (3) We always actively encourage young people to involve family members and/or significant others from assessment onwards. However, this is not usually revisited if the person initially declines involvement.<br>• (2) We always actively encourage young people to involve family members and/or significant others to join the assessment. We do not routinely encourage involvement in treatment. This is not revisited if the person initially declines involvement.<br>• (1) We may sometimes (but not always) ask young people to invite family members or significant others to join the assessment, and do not actively encourage/push this. They have no focused involvement in treatment. | | |
| 32 | **Age-related transitions** | Interview | • (5) We are an all-age, 0-25, or age-integrated service.<br>• (4) Coordinated, planned transitions within the conventional CAEDS-AEDS model. Transitions | 2.27% | |

| Item # | Criterion | How assessed | Scoring | Weight | Score |
|---|---|---|---|---|---|
| | | | are overseen by a dedicated clinician or group of clinicians, and are flexible, person-centred and needs-based (e.g., may involve completing the course of treatment started in CAEDS or developing a new plan in AEDS as per patient and family circumstances).<br>• (3) Flexible transition age within conventional CAEDS-AEDS model and we try to plan transitions in advance and work closely with CAEDS colleagues.<br>• (2) We operate within the conventional CAEDS-AEDS model with a transition at age 18. However, we try to plan transitions in advance and work closely with CAEDS colleagues.<br>• (1) We operate within the conventional CAEDS-AEDS model with a strict transition at age 18. We try to but are not consistent with planning transitions in advance with patients and are unable to plan closely with CAEDS colleagues. | | |
| 33 | **University transitions** | | • (5) Service carefully prepares for transitions to university using individualised care plans and (where possible) joint care plans with other institutions (e.g., GP, university services) to provide specialised care for student patients registered in two catchment areas. Service has the option to keep and not discharge | 2.27% | |

| Item # | Criterion | How assessed | Scoring | Weight | Score |
|---|---|---|---|---|---|
| | | | patients until they are settled in a new service.<br>• (4)<br>• (3) Service does not consistently use individual care plans or make use of a joint care approach where possible, and discharges patients who have moved out of catchment area for university.<br>• (2)<br>• (1) Service provides university transition preparation resources only, with no planning coordination. | | |
| 34 | **Community awareness and education;<br>Outreach (to schools, GPs, etc.)** | Interview | • (5) Extensive evidence of outreach and education activities to multiple organisations / groups in the community (e.g., universities, GPs, schools, charities) on a regular basis (e.g., >=3 x year).<br>• (4) Evidence of outreach and education to multiple organisations / groups in the community, but on an irregular basis (up to 1-2x year).<br>• (3) Evidence of outreach and education but this is limited in scope, i.e., to GPs only or universities only. Outreach to this one sector occurs regularly.<br>• (2) Evidence of outreach and education but this is limited in scope, i.e., to GPs only or universities only. Outreach to this one sector occurs on an irregular basis. Or, outreach practices are limited but FREED Champion is known in the area and available for consultation. | 2.27% | |

| Item # | Criterion | How assessed | Scoring | Weight | Score |
|---|---|---|---|---|---|
| | | | • (1) No or very little evidence e.g., prior one-off example of community awareness and education / outreach, which has not been repeated. | | |
| 35 | **Diversity, inclusion of and adaptations for under-served groups.** These include: Improved diverse practices in recruitment, and more representative staffing Adapted, enhanced, or specific treatment pathways to accommodate for specific additional needs (e.g., PEACE pathway for autistic people) Ensuring service environment accommodates for all people (e.g., modifying the built environment – chairs to accommodate for all weights) Ensuring diversity in service resources (e.g., website shows people of varied body sizes and/or ethnicities, leaflets in multiple languages) | | • (5) Evidence of improvements or established practices to promote diversity and inclusivity in the service. These span all areas including: recruitment practices and equal representation in staffing; adaptations to or specific treatment pathways; an inclusive service environment and representation. In addition, evidence of conducting research/ evaluation activities in this area (e.g., focus groups/interviews with service users, diversity tracker data analysis).<br>• (4)<br>• (3) Evidence of multiple improvements or established practices to promote diversity and inclusivity in service, or extensive training for FREED Champion/mini-team. These improvements span more than one of the areas above but not all.<br>• (2)<br>• (1) No or very limited evidence e.g., of only one attempt to improve diversity and inclusivity in the service. This may be a single targeted attempt in only one of the above example areas or may include one-off relevant training. | 2.27% | |

***Note***. FREED – First Episode Rapid Early Intervention for Eating Disorders; DUED – Duration of Untreated Eating Disorder; ED – Eating Disorder; NICE – National Institute for Health and Care Excellence; CBT – Cognitive Behavioural Therapy; MANTRA - Maudsley Model of Anorexia Nervosa Treatment for Adults; SSCM – Specialist Supportive Clinical Management; ARFID – Avoidant Restrictive Food Intake Disorder; AN – Anorexia nervosa; BN – Bulimia nervosa; WTE – whole-time equivalent; KCL – King's College London; FT-AN – Family Therapy for Anorexia Nervosa; CAEDS – Child and Adolescent Eating Disorder Service; AEDS – Adult Eating Disorder Service; PEACE - Pathway for Eating disorders and Autism developed from Clinical Experience.
